# Novel Amplitude-Based Approach for Reducing Sidelobes in Persistent Scatterer Interferometry Processing Using Spatially Variant Apodization

**DOI:** 10.3390/s26010204

**Published:** 2025-12-28

**Authors:** Natascha Liedel, Jonas Ziemer, Jannik Jänichen, Christiane Schmullius, Clémence Dubois

**Affiliations:** 1 Department for Earth Observation, Friedrich Schiller University Jena, Leutragraben 1, 07743 Jena, Germany; jonas.ziemer@uni-jena.de (J.Z.); jannik.jaenichen@uni-jena.de (J.J.); c.schmullius@uni-jena.de (C.S.); 2 German Aerospace Center, Institute of Data Science, Mälzerstraße 3, 07743 Jena, Germany; clemence.dubois@dlr.de

**Keywords:** SAR, S-1, PSI, sidelobes, SVA, StaMPS, SNAP2StaMPS, active reflector, amplitude-based filtering, dam monitoring

## Abstract

This study introduces an amplitude-based method that applies Spatially Variant Apodization (SVA) to reduce sidelobes in Synthetic Aperture Radar (SAR) data. Unlike conventional approaches, the filter is applied only to the amplitude while preserving the original interferometric phase, thereby enabling accurate Persistent Scatterer Interferometry (PSI) for dam deformation monitoring in Stanford Method for Persistent Scatterers (StaMPS) software. The SVA filter is integrated as an additional processing step within the Sentinel Application Platform (SNAP) for the SentiNel Application Platform to Stanford Method for Persistent Scatterers (SNAP2StaMPS) workflow. Filtering is performed in two dimensions (azimuth and range) separately on the In-phase (I) and Quadrature (Q) components of the coregistered data using a Python-based implementation via SNAP-Python (snappy). By recombining the SVA-filtered and original I and Q components, the method modifies only the amplitude while leaving the phase unchanged. The approach is evaluated in a proof-of-concept case study of the Sorpe Dam in Germany, where an Electronic Corner Reflector - C Band (ECR-C) produces sidelobe artifacts that degrade the Sentinel-1 (S-1) descending time series. The results demonstrated a successful integration of SVA filtering into the SNAP2StaMPS framework, achieving sidelobe reduction and improved Persistent Scatterer (PS) detection without compromising phase quality. The number of sidelobe-affected PS decreased by 39.26% after SVA filtering, while the amplitude-based approach preserved the original phase and deformation values, with a Root Mean Square Error (RMSE) of approximately 0.38 mm. Overall, this novel amplitude-based SVA approach extends the SNAP2StaMPS workflow by reducing strong sidelobes while preserving phase information for dam monitoring at the Sorpe dam site.

## 1. Introduction

Synthetic Aperture Radar (SAR) is a powerful remote sensing technique that enables Earth observation independently of weather conditions and daylight by transmitting microwave pulses and recording their backscattered echoes from the Earth’s surface [1]. Interferometric Synthetic Aperture Radar (InSAR) measures surface deformation and topography by comparing the phase information of at least two SAR images acquired at different times or from slightly different positions [2]. Among interferometric methods, Persistent Scatterer Interferometry (PSI) identifies long-term coherent scatterers, known as Persistent Scatterer (PS), that maintain phase stability over time. This makes PSI particularly effective in urban environments, where human-made structures such as dams serve as stable objects. By analyzing phase differences across multiple SAR acquisitions over the same area, PSI achieves millimeter-level accuracy in deformation measurements [3,4,5]. The Sentinel-1 (S-1) SAR mission, with its 6 to 12 day repeat cycle and Line of Sight (LOS) imaging geometry, is particularly well-suited for long-term monitoring using PSI [6].

**Figure 1:**
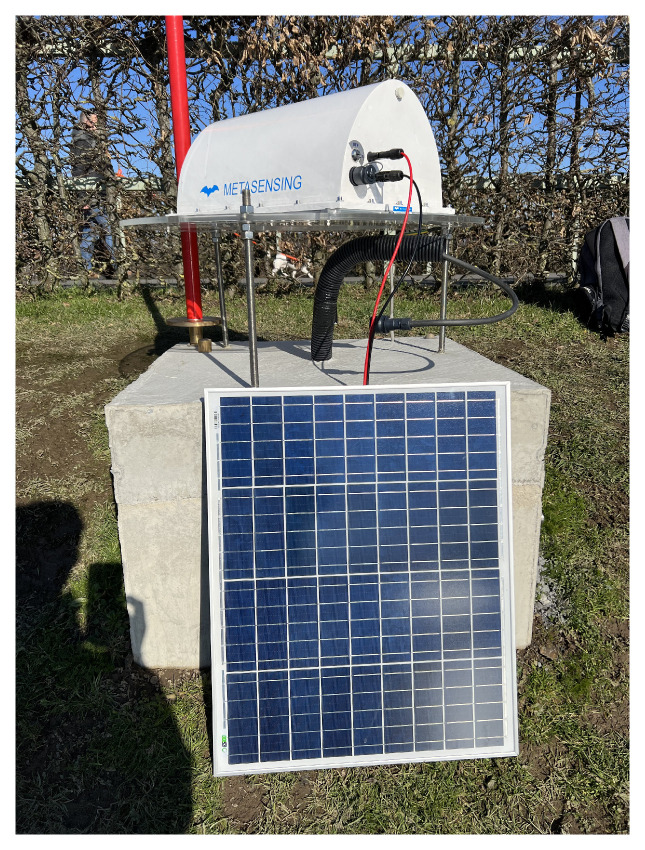
Electronic Corner Reflector—C band (ECR-C) [11].

**Figure 2:**
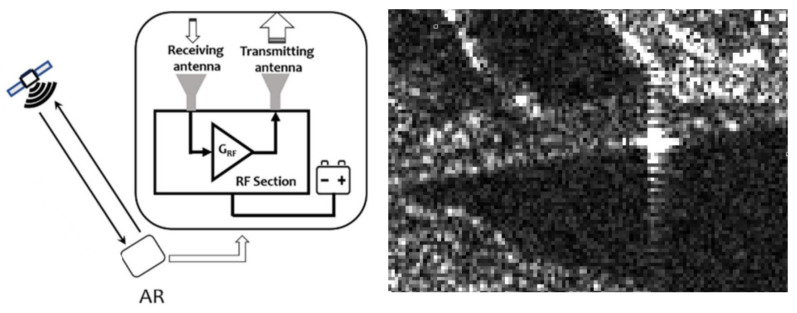
**Left**: Working principle of Active Reflectors (ARs) [8]. **Right**: Amplitude Image showing the Sidelobe around the Sorpe Dam, Germany (13 May 2023).

Corner Reflectors (CRs) enhance PSI applications by providing highly stable reference points. At the study site, an Electronic Corner Reflector—C band (ECR-C) is installed for use with C-band SAR systems such as S-1, which operates at a frequency of 5.4 Gigahertz (GHz) with a bandwidth of up to 100 Megahertz (MHz) [7]. The installed ECR-C is shown in Figure 1. This Active Reflector (AR), enables precise deformation monitoring with millimeter-level accuracy and actively interacts with SAR signals by responding to transmitted pulses and amplifying the returned echoes [8,9]. The working principle of ARs is illustrated in Figure 2 (left). An AR consists of a receiving antenna, an amplification stage, and a transmitting antenna. The signal arriving from the satellite is first received, then amplified, and finally re-transmitted back toward the same satellite [8]. However, despite these advantages, their strong backscatter can introduce sidelobe artifacts, which appear as bright star-like cross patterns in SAR data [10], as shown in Figure 2 (right), where the sidelobe arises in the study area at the location of the ECR-C signal.

**Figure 3:**
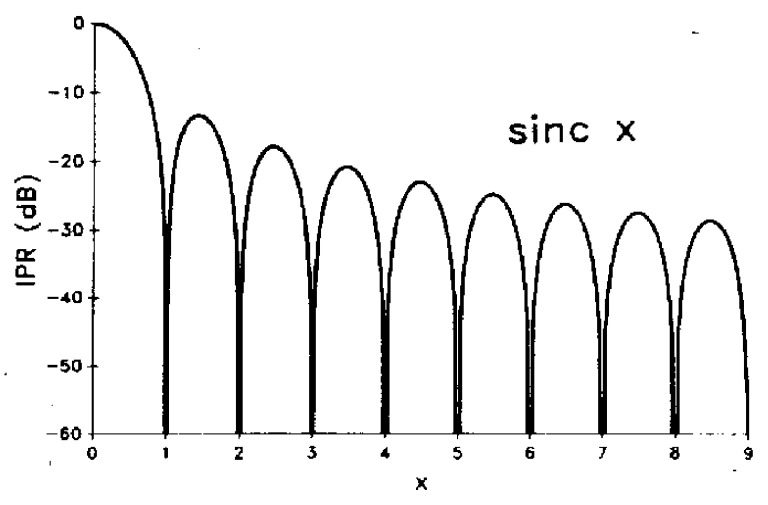
Unweighted (sinc) Impulse Response (IPR) of a Synthetic Aperture Radar (SAR) system [14].

Sidelobes occur because energy is radiated not only in the mainlobe of the SAR antenna but also unintended directions [1]. They are described by the system’s Impulse Response (IPR), which characterizes how a point target appears in the SAR image. A typical IPR follows a sinc pattern, featuring a sharp mainlobe surrounded by diminishing sidelobes [1,12]. The sinc function, illustrated in Figure 3, features a dominant central peak (mainlobe) that contains most of the SAR signal energy, with decibel (dB) values indicating how much weaker the sidelobes are compared to the mainlobe [13]. The width of the mainlobe determines image resolution: a narrower mainlobe yields higher resolution, while a wider mainlobe reduces detail. The oscillations extending outward from the mainlobe are known as sidelobes [14]. Sidelobes arise because a rectangular aperture in the spatial domain transforms into a sinc function in the frequency domain. This rectangular structure allows for precise identification of sidelobes in each pixel and enables selective enhancement of the mainlobe [15]. However, sidelobes may distort or obscure surrounding signal information, leading to potential misinterpretations in PSI-derived deformation time series [10]. Although careful system design and advanced filtering techniques can mitigate these effects, sidelobes cannot be entirely eliminated [1]. Therefore, sidelobe reduction constitutes a crucial preprocessing step in SAR image analysis [10].

## 2. State of the Art and Research Gap

### 2.1. Sidelobe Suppression with Apodization Techniques

Apodization refers to the application of weighting functions to the SAR signal in order to control the shape of the system’s IPR. Various techniques have been developed to reduce sidelobe artifacts in SAR imagery. Linear apodization methods such as Hamming, Hanning and Blackman windows apply fixed weighting functions to reduce sidelobes but compromise image resolution through mainlobe broadening [16,17,18]. A comparative analysis of linear apodization methods can be found in Podder et al. (2014) [17]. Non-linear apodization approaches like Dual Apodization (DA), Complex Dual Apodization (CDA), Tri-Apodization and Spatially Variant Apodization (SVA) offer more advanced sidelobe reduction while preserving resolution by dynamically adjusting weighting based on local image characteristics rather than applying a uniform function across the entire image [14,19,20,21]. Among these, SVA is the most extensively studied in the literature due to its ability to adapt filter weights on a per-pixel basis, assigning each pixel an individual weighting function to optimally reduce sidelobe interference [22,23]. Stankwitz et al. provided an explanation and comparison of linear and non-linear apodization techniques in the cited reference [14].

According to Fischer et al. (2006) [15], optimal sidelobe reduction with SVA is achieved when the SAR data (1) exhibit a rectangular spectral shape, (2) are sampled at the Nyquist rate or an integer multiple thereof, and (3) are free from spectral shifts such as those caused by squint. Further explanations are provided in the cited reference, as these details go beyond the scope of this study. Recent developments extend the concept of SVA. Robust Spatially Variant Apodization (RSVA) enhances both sidelobe reduction and resolution, allowing for the detection of weaker targets, albeit at the cost of increased computational complexity [24]. General Spatially Variant Apodization (GSVA) and Modified Spatially Variant Apodization (MSVA) further broaden the applicability of SVA, making it effective under diverse sampling conditions and thereby reducing preprocessing time [25]. Emerging hybrid models integrate SVA with machine learning and compressive sensing techniques, further improving sidelobe reduction performance under complex and variable imaging scenarios [25,26].

However, the application of SVA in the context of InSAR remains relatively unexplored. Existing studies show that when applied to interferometric image pairs, SVA enhances image resolution and improves phase accuracy by increasing coherence between acquisitions, but at the same time degrades phase information [22]. Furthermore, SVA enables a more targeted selection of PS candidates. Chaabane et al. (2009) [13] found that the PS points detected after SVA filtering are also present in the unfiltered data. However, the filtered approach demonstrates greater selectivity. A further advancement by Iglesias & Mallorqui (2013) [23] and Iglesias et al. (2014) [27] involved using SVA only to identify pixels affected by sidelobes to create a Sidelobe Risk Mask (SLRM). The SLRM marks the affected areas to exclude them from further filtering, ensuring that only reliable pixels contribute to the final InSAR analysis. Nevertheless, the use of a SLRM does not exploit SVA’s potential as a direct image-domain filter, while applying SVA directly to interferometric data entails the risk of phase degradation, which is critical for phase-sensitive applications such as PSI.

### 2.2. Research Approach

To address this research gap, this paper presents the journal-level continuation of the first author’s Master’s thesis, completed under her former name, Stumpf (2025) [28]. SVA is applied directly to SAR imagery, and a novel amplitude-based SVA approach is introduced to preserve phase integrity. The focus on amplitude is motivated by the PSI software Stanford Method for Persistent Scatterers (StaMPS) (Version StaMPS-4.1-beta), which performs an initial selection of PS candidates using the amplitude dispersion index [29]. By suppressing sidelobe-related PS candidates at this stage, their propagation into subsequent phase-driven analyses is prevented. The SVA filter is tested during PSI processing on a dam equipped with an ECR-C, which produces strong sidelobes that lead to false PS detections in surrounding areas. SVA is integrated into the established S-1 PSI workflow via SentiNel Application Platform to Stanford Method for Persistent Scatterers (SNAP2StaMPS), combining preprocessing in Sentinel Application Platform (SNAP) with further PSI analysis in StaMPS software. The novel amplitude-based SVA approach is applied only to the amplitude, while the original phase information remains unchanged. To achieve this, the complex SAR signal is recombined after filtering, ensuring that sidelobe reduction does not compromise phase coherence.

In the literature, sidelobe reduction approaches are commonly referred to as sidelobe suppression. In this work, the term reduction is used deliberately, since the objective is not to develop a perfect sidelobe suppression technique for SAR analysis in general, but rather to identify a method optimized for this specific case, while the SVA refinements outlined in Section 2.1 offer promising capabilities, they are not applied here due to their implementation complexity. Furthermore, the sampling conditions for SVA are not adjusted, as this lies beyond the scope of the study. The S-1 data are used as acquired and are provided in zero-Doppler geometry, thereby fulfilling the preconditions for successfully applying the SVA filter as described in Fischer et al. [15].

The integration of the SVA method into the established PSI workflow, along with testing the application of SVA to amplitude only, is expected to reduce false PS detections. The amplitude-based approach is anticipated to produce results closer to the original output, as the original phase is preserved. Overall, this study aims to evaluate SVA in general for PSI processing and to test the proposed novel amplitude-based SVA approach.

## 3. Materials and Methods

### 3.1. Study Area

**Figure 4:**
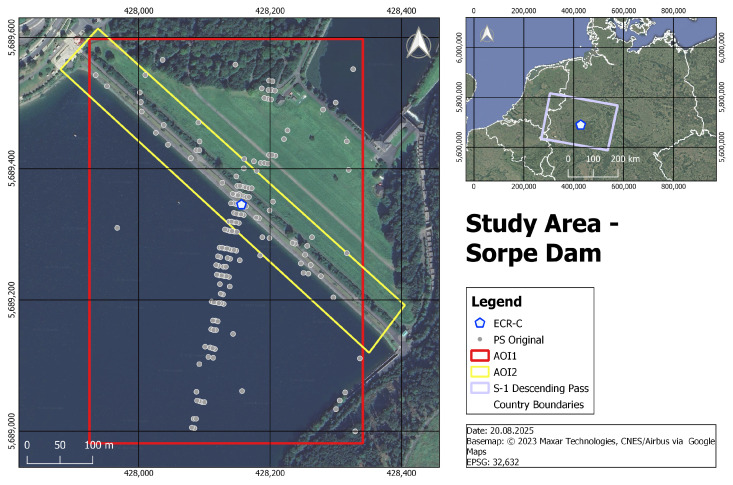
Study Area and Persistent Scatterer (PS) Distribution at Sorpe Dam.

The Sorpe Dam serves as the study area for this case study. It is an earth-fill dam that was constructed between 1926 and 1935 by the Ruhrtalsperrenverein in the Sauerland region, Germany. The dam has a full storage capacity of approximately 70 million cubic meters, with a full reservoir level at 283.30 m above sea level and a crest height of 285.60 m above sea level. At full capacity, the reservoir surface area covers around 3.3 square kilometers. For flood control, the dam features a high-water discharge system consisting of an overflow weir with cascades and a single floodgate with a discharge capacity of 100 cubic meters per second [30]. The extent of the dam is shown in the left map of Figure 4.

At various Ruhrverband dam sites, ECR-Cs are installed to enable dam monitoring using PSI, with the broader objective of developing an automated, satellite-based deformation monitoring system. The methodology for site identification and ECR-C setup can be reviewed in Jänichen et al. (2025) [31]. Of all the dams managed by the Ruhrverband, the Sorpe Dam exhibits the most pronounced sidelobe effects. This makes it an ideal scenario for testing sidelobe reduction in PSI analysis and can be attributed to the combination of the dam’s orientation relative to the satellite overpass (see Section 3.2) and the working principle of the ECR-C (see Section 1). The location of the Sorpe Dam, including the position of the ECR-C, is shown in the right-hand overview map in Figure 4, while the left-hand map depicts the exact location of the ECR-C on the Sorpe Dam crest. The grey dots on the left map represent PS candidates detected in the dataset without the use of sidelobe reduction techniques. Each PS point contains deformation information for its specific position in the study area over the entire time series. The impact of the sidelobes is visible in the form of false PS points extending almost 350 m into the water, away from the dam crest. Furthermore, two Areas of Interest (AOIs) are defined to evaluate the effectiveness of sidelobe reduction. AOI1 (red) serves as a bounding box to validate the spatial extent of PS across the entire dam site, whereas AOI2 (yellow) focuses on PS located on the dam crest, which are assumed to reflect actual dam deformation. The AOIs are not intended to be strictly perpendicular but to allow assessment of impacts on other potential natural PS occurring on the dam.

### 3.2. Data

The S-1 mission is the European Radar Observatory under the Copernicus initiative, a collaboration between the European Commission and the European Space Agency (ESA). It consists of two sun-synchronous, polar-orbiting satellites with an orbital phase difference of 180°, operating in C-band SAR imaging. Each S-1 satellite follows a near-polar orbit with a 12 day repeat cycle. Since ascending and descending passes alternate, the same ground track is revisited every 6 days by the satellite constellation [32]. Due to its right-looking geometry, S-1 observes the Earth’s surface from east to west in descending mode and from west to east in ascending mode [6]. On 23 December 2021, one of the satellites, S-1B, was declared ended due to an anomaly in the instrument’s power supply, which rendered it unable to deliver SAR data. Following the loss of S-1B, the observation scenario of the remaining satellite, S-1A, was adjusted to a 12-day repeat cycle under a single-satellite constellation. This configuration remained in place until 5 December 2024, when S-1C was launched into orbit as the successor to S-1B [32].

This study utilizes 20 S-1 Level-1 products acquired in Interferometric Wide Swath (IW) mode using the Terrain Observation by Progressive Scans Synthetic Aperture Radar (TOPSAR) technique. The Single Look Complex (SLC) scenes were captured between 13 May 2023 and 27 December 2023 during descending passes at 05:42 AM, when only S-1A was operational, following a 12-day repeat cycle. The chosen time frame corresponds to the operational period of the ECR-C, during which no settings were changed. The dataset was obtained from the Alaska Satellite Facility [33]. Figure 4 (right) illustrates the spatial extent of the S-1 descending pass acquisitions, where the purple outline marks the coverage area. Descending passes are selected because they exhibit more pronounced sidelobes than ascending passes in the study area. This can be attributed to the specific observation geometry as depicted in Section 3.1. The S-1 descending pass moves from east to west, while the dam crest is oriented from southeast to northwest. This combination of geometric alignment and ECR-C’s signal amplification produce strong backscatter, resulting in the pronounced sidelobes observed in the SAR imagery.

In addition, this study uses the 1 arc-second global Digital Elevation Model (DEM) from the Shuttle Radar Topography Mission (SRTM), conducted by the National Aeronautics and Space Administration (NASA) and the National Geospatial-Intelligence Agency. This dataset is automatically downloaded via the SNAP2StaMPS workflow within SNAP, using the ESA Scientific Toolbox Exploitation Platform (STEP) repository. The DEM is employed during PSI preprocessing for coregistration, aligning the reference and secondary S-1 SLC split products based on their orbital metadata and the SRTM DEM (see Section 4).

### 3.3. Software

This study uses SNAP, developed by the ESA, an open-source toolbox for processing SAR and optical remote sensing data. SNAP (Version 9) is used for preprocessing S-1 data following the SNAP2StaMPS workflow for further PSI processing [34].

The SNAP2StaMPS workflow, developed by Blasco et al. (2019) [35], prepares TOPSAR data in SNAP for seamless integration with the StaMPS PSI processing workflow. SNAP2StaMPS is open-source, and available on GitHub (Version 1) [36]. The workflow is structured around SNAP processing graphs and a set of Python scripts that serve as a wrapper, allowing users to configure key processing parameters through a configuration file and execute the workflow automatically. This approach supports batch processing of single-reference interferogram stacks, where users define parameters such as the project folder, subswath selection, and bounding box coordinates of the AOI [35].

StaMPS (Version StaMPS-4.1-beta), developed by Hooper et al. (2012) [37], is a MATrix LABoratory (MATLAB)-based software package for PSI processing. StaMPS processes the stacks of interferograms generated by SNAP and applies a statistical approach to identify stable PS for displacement monitoring. The software is compatible with the Toolbox for Reducing Atmospheric InSAR Noise (TRAIN) developed by Bekaert et al. (2015) [38] and the Statistical-Cost Network Flow Algorithm for Phase Unwrapping (SNAPHU), introduced by Chen & Zebke (2001) [39].

This study uses two versions of Python due to software compatibility constraints. SNAP Version 9 requires Python 2.7.16, while all other Python-based processes run on Python 3.10.16 [40]. In addition, the SNAP-Python (snappy) interface is used to ensure compatibility between the custom Python program and SNAP. This ESA snappy plugin is an internal component of SNAP, automatically installed during software setup and implemented by Brockman Consult Scientific Image Processing Toolbox [41].

### 3.4. Cartesian and Polar Representations of SAR Pixels

**Figure 5:**
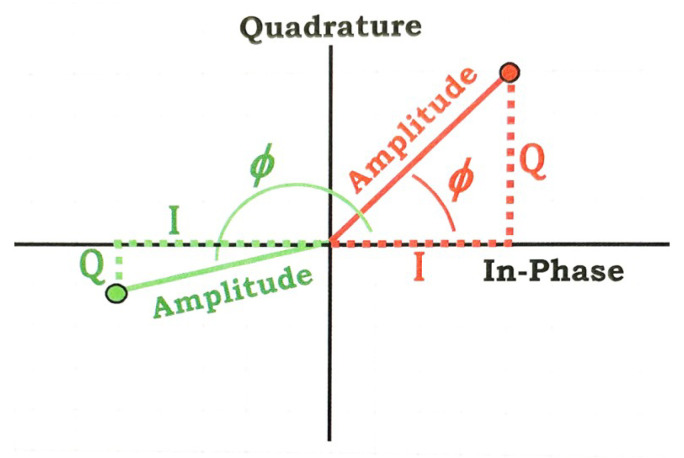
Relationship between Cartesian In-phase (I) and Quadrature (Q) and Polar Amplitude (A) and Phase (ϕ) Representations [1].

After image formation, the final SAR product is represented as complex SAR image with two components: The In-phase (I) component represents the real part of the signal, while the Quadrature (Q) component corresponds to the imaginary part. The relationship between the Cartesian (I, Q) and polar (amplitude, phase) representations is illustrated in Figure 5 and is given by:

(1) $$\mathrm{Amplitude}=\sqrt{I^{2}+Q^{2}}$$

(2) $$\mathrm{Phase}=\arctan\left(\frac{Q}{I}\right)$$

(3) I=Amplitude×cos(ϕ)

(4) Q=Amplitude×sin(ϕ)

Here, cos(ϕ) and sin(ϕ) describe the geometric projection of the amplitude onto the respective axes: cos(ϕ) represents the share of the signal along the I axis, while sin(ϕ) corresponds to the share along the Q axis. Thus, cos(ϕ) gives the I component aligned with the reference wave, and sin(ϕ) gives the Q component shifted by $90^{{}^{\circ}}$, while amplitude and phase provide an intuitive understanding of SAR signals, the I and Q components simplify computations [1]. This relationship is relevant for understanding the novel amplitude-based approach, described in this paper, which preserves the original phase by recombining the I and Q components, while applying SVA filtering exclusively to the amplitude (Section 3.6).

### 3.5. Spatially Variant Apodization (SVA)

SVA, first introduced by Stankwitz et al. (1994) [14] reduces sidelobes by identifying pixels at the peak of the mainlobe and reducing sidelobes surrounding them. Typically, the SVA uses a three-point convolution, where each pixel is influenced by its own value and the values of its two neighbors [13,14]. SVA adopts cosine-on-pedestal weighting where the cosine-on-pedestal parameter *w* changes from 0 to 0.5. The choice of the weighting function (e.g., Hamming, Hanning) directly controls the trade-off between mainlobe width and sidelobe suppression. These windows correspond to fixed values of *w* (e.g., w=0 for rectangular, w=0.5 for Hanning, w≈0.43 for Hamming). In contrast, SVA does not rely on a predefined window function. Instead, it evaluates all possible weighting values in the range 0≤w≤0.5 and selects, for each pixel, the value that minimizes the output energy [42].

The SVA method applied in this study extended the existing approach by Wang et al. (2012) [42] to two-dimensional (2D) processing, incorporating both azimuth and range directions. Thereby, SVA was applied separately to the I and Q components of each SAR image. The adapted 2D weighting function was defined as follows:

(5) $$Iw_{u}\left(m,n\right)=\frac{-I(m,n)}{I(m-1,n)+I(m+1,n)+I(m,n-1)+I(m,n+1)}$$

(6) $$Qw_{u}\left(m,n\right)=\frac{-Q(m,n)}{Q(m-1,n)+Q(m+1,n)+Q(m,n-1)+Q(m,n+1)}$$

Here $w_{u}\left(m,n\right)$ represented the weighting factor for pixel (m,n), computed using its four direct neighbors. If any of these neighboring pixels contained NaN values, they were excluded from the computation. The threshold of 0.5 follows directly from the cosine-on-pedestal weighting theory used in canonical 1D SVA [42]. The proposed 2D implementation generalizes the original 1D stencil to the smallest symmetric 2D neighbourhood (four direct neighbours) while preserving the theoretical constraints on *w*. Using the computed weighting function, the final SVA filtering was applied as follows:

(7) $$I_{w}\left(m,n\right)=\left\{\begin{array}{cc} I(m,n), & w_{u}\left(m,n\right)<0\\ 0, & 0\leq w_{u}\left(m,n\right)\leq \frac{1}{2}\\ I\left(m,n\right)+\frac{1}{4}\left[I(m-1,n)+I(m+1,n)+I(m,n-1)+I(m,n+1)\right], & w_{u}\left(m,n\right)>\frac{1}{2} \end{array}\right.$$

(8) $$Q_{w}\left(m,n\right)=\left\{\begin{array}{cc} Q(m,n), & w_{u}\left(m,n\right)<0\\ 0, & 0\leq w_{u}\left(m,n\right)\leq \frac{1}{2}\\ Q\left(m,n\right)+\frac{1}{4}\left[Q(m-1,n)+Q(m+1,n)+Q(m,n-1)+Q(m,n+1)\right], & w_{u}\left(m,n\right)>\frac{1}{2} \end{array}\right.$$

Finally, the SVA filtering process was applied using the following implementations:

- If $w_{u}\left(m,n\right)<0$, the mainlobe remained unchanged, meaning the pixel retained its original value.
- If $0\leq w_{u}\left(m,n\right)\leq \frac{1}{2}$, sidelobes were reduced by setting the pixel value to zero.
- If $w_{u}\left(m,n\right)>\frac{1}{2}$, the pixel value was replaced by the mean of its four valid neighboring values.

### 3.6. Novel Amplitude-Based Approach

The proposed amplitude-based method built upon SVA filtering. First, SVA was applied to the I and Q components as described in Section 3.5, producing a sidelobe-reduced version of the scene. In a second step, new I and Q components were recalculated by combining the phase information from the original data (before SVA-filtering) with the amplitude of the SVA-filtered data. The modified I and Q components were given by:

(9) $$I_{\mathrm{modified}}=I_{\mathrm{orig}}\times \frac{\sqrt{I_{\mathrm{reduc}}^{2}+Q_{\mathrm{reduc}}^{2}}}{\sqrt{I_{\mathrm{orig}}^{2}+Q_{\mathrm{orig}}^{2}}}$$

(10) $$Q_{\mathrm{modified}}=Q_{\mathrm{orig}}\times \frac{\sqrt{I_{\mathrm{reduc}}^{2}+Q_{\mathrm{reduc}}^{2}}}{\sqrt{I_{\mathrm{orig}}^{2}+Q_{\mathrm{orig}}^{2}}}$$

Here, *orig* referred to the original unfiltered data, and *reduc* represented the sidelobe-reduced counterpart. These expressions were derived from the relationships between cartesian (I, Q) and polar (amplitude, phase) representations discussed in Section 3.4, ensuring that the original phase was preserved while the amplitude was modified to reduce sidelobes using the SVA filter.

## 4. Workflow

The workflow followed the SNAP2StaMPS and StaMPS approaches described by Blasco et al. (2019) [35] and Hooper et al. (2018) [29], respectively. SNAP2StaMPS was used to prepare the time series of S-1 SLC images within SNAP for subsequent PSI analysis in the StaMPS software. An additional sidelobe reduction step was introduced by integrating the SVA filter into the SNAP2StaMPS workflow and the novel amplitude-based approach by applying SVA only to the SAR amplitude while preserving the original phase. The StaMPS processing chain remained unchanged. Table A1 summarizes the specific SNAP2StaMPS settings used for this case study based on the characteristics of the study area. The processing parameters for StaMPS were set to their default values, except for those listed in Table A2, which were selected based on the study area’s characteristics or informed by Ziemer et al. (2025) [11] in the context of dam monitoring.

### 4.1. Standard Preprocessing with SNAP2StaMPS

**Figure 6:**
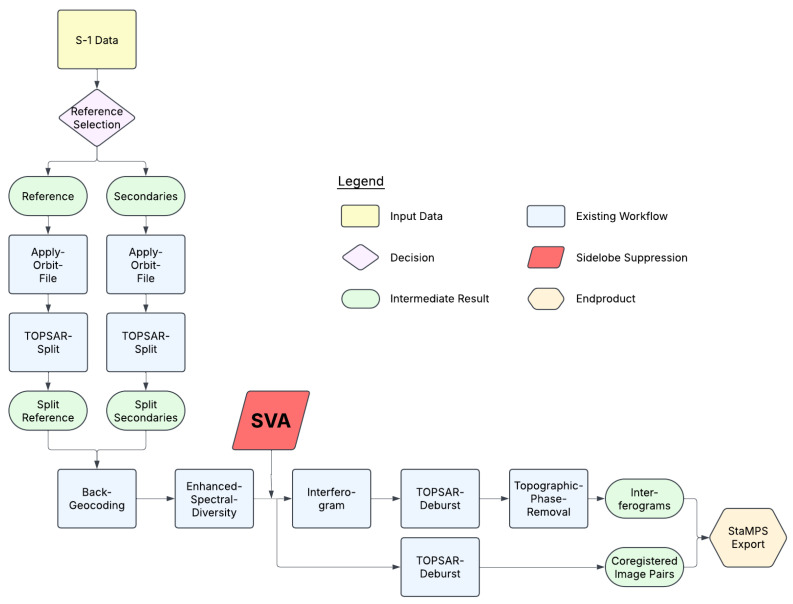
SentiNel Application Platform to Stanford Method for Persistent Scatterers (SNAP2StaMPS) Workflow with integrated Spatially Variant Apodization (SVA) filter for Sidelobe Reduction.

The traditional SNAP2StaMPS workflow is described by Blasco et al. (2019) [35]. Detailed explanations of the SNAP2StaMPS processing are available in the cited reference. Figure 6 illustrates the traditional SNAP2StaMPS workflow, with the integrated SVA filter highlighted in red. The SNAP2StaMPS processing chain begins with the selection of a reference scene from the entire time series, represented by the purple diamond shape. Then, the TOPSAR-Split and Apply-Orbit-File steps are performed separately for both the reference and secondary products. These steps are automated using a predefined processing graph within SNAP2StaMPS. Coregistration and interferogram computation are performed using a second processing graph, which executes both steps in a single process. The Back-Geocoding operator coregisters two S-1 SLC split products (reference and secondary) by aligning them using their orbital metadata and the SRTM 1 arc-second global DEM. Following Back-Geocoding, the Enhanced-Spectral-Diversity step estimates a constant range offset for the entire subswath using incoherent cross-correlation to determine a final correction, ensuring complete coregistration. At this stage, the workflow splits into two branches, each leading to different intermediate outputs. In the first branch, the next step is Interferogram Generation, where the complex interferogram is computed. This is followed by the TOPSAR-Deburst step, which merges bursts into a continuous image. The second processing branch follows the same workflow but terminates at this stage. In the first processing branch, an additional step, Topographic-Phase-Removal, eliminates the topographic phase by converting the SRTM 1 arc-second global DEM into SAR coordinates and subtracting the corresponding phase from the interferogram. Upon completion of this graph, the two intermediate outputs, Interferograms and Coregistered Image Pairs (highlighted in green), are generated, concluding this stage of the workflow. The final step of the SNAP2StaMPS workflow, StaMPS Export, prepares the data in a format compatible with StaMPS.

### 4.2. Standard PSI Processing with StaMPS

The methodology of the StaMPS workflow is described by Hooper et al. (2018) [29]. Detailed explanations of the StaMPS processing are available in the cited reference. The StaMPS export products generated by the SNAP2StaMPS workflow (Section 4.1) serve as input data for this stage. Before initiating PSI processing with StaMPS, an initial selection of PS candidates is performed using the amplitude dispersion index. PSI processing is then carried out in the MATLAB environment (Version R2024a), following steps 1 to 7 and utilizing the TRAIN software (Version 3beta), as described by Hooper et al. (2018) [29]. Temporal coherence is estimated during processing, and pixels are selected probabilistically based on their noise characteristics by comparing observed data to randomly generated phase data. The final outcome of StaMPS is a set of PS candidates with coordinates and deformation time series.

### 4.3. Implementation of SVA Within the SNAP2StaMPS Workflow

SVA filtering was applied after the coregistration following Back-Geocoding and Enhanced Spectral Diversity (Figure 6). SVA operates on complex SAR data on a line-by-line basis. Its effectiveness depends on accurately aligned input. Coregistration ensures that reference and secondary acquisitions are geometrically consistent (see Section 4.1). Therefore, SVA was applied immediately after coregistration, allowing the filter to operate on data that are both geometrically aligned and as close to the raw acquisitions as possible [15]. As SNAP does not natively support SVA, the filtering was implemented externally via a custom Python script using the snappy interface. The SVA implementation followed the methodology developed in this work by Liedel (2025) [43], as described in Section 3.5, and is publicly available as Python code on GitHub. To incorporate the SVA filter, the second graph of the traditional SNAP2StaMPS workflow was split into two parts, enabling the external application of the SVA filter in Python between the coregistration and interferogram generation stages.

### 4.4. Implementation of Novel Amplitude-Based Approach Within the SNAP2StaMPS Workflow

**Figure 7:**
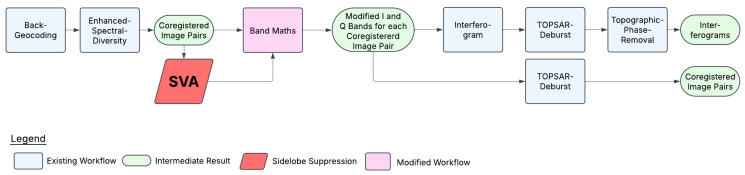
Second Graph of SNAP2StaMPS Workflow with integrated Amplitude-based SVA filter for Sidelobe Reduction.

The rationale of the novel amplitude-based approach was that StaMPS initially selects PS candidates based on the amplitude dispersion index, while phase characteristics are only incorporated during PSI processing (Section 4.2). By applying SVA filtering exclusively to the amplitude, sidelobe-affected pixels were reduced and false PS candidates were minimized, while the original phase was preserved to ensure reliable PS selection based on phase information. To implement the amplitude-based approach, the traditional second SNAP2StaMPS processing graph was restructured into a three-step procedure, illustrated in Figure 7, with the intermediate steps highlighted in green. In the first step, standard processing was executed up to sidelobe reduction. SVA filtering was then applied via the snappy interface, as described in Section 4.3. At this stage, both the original coregistered (unfiltered) data and the SVA-filtered data were stored for each scene, since both are required for the amplitude-based processing. In the second step, the Band Maths tool in SNAP (highlighted in magenta) implemented the amplitude-based approach: for each scene, Band Maths loaded both the original and SVA-filtered data and recombined the original phase with the SVA-filtered amplitude by recalculating the I and Q components, following the procedure in Section 3.6. Band Maths was chosen because it is available within SNAP, minimizing the need to outsource operations to external software. Finally, after the Band Maths step, the second processing graph resumed, continuing the standard SNAP2StaMPS workflow.

## 5. Results and Discussion

### 5.1. Results

To evaluate the effectiveness of the proposed methods, three processing variants were analyzed: (1) the original processed data, abbreviated as Original; (2) data with conventional SVA filtering applied to the entire scene, affecting both amplitude and phase (SVA_entireScene); and (3) data with SVA filtering applied only to the amplitude, preserving the original phase (SVA_amplitudeBased). For comparison, the spatial distribution of PS points, deformation patterns, IPRs, and amplitude and phase characteristics were examined.

**Figure 8:**
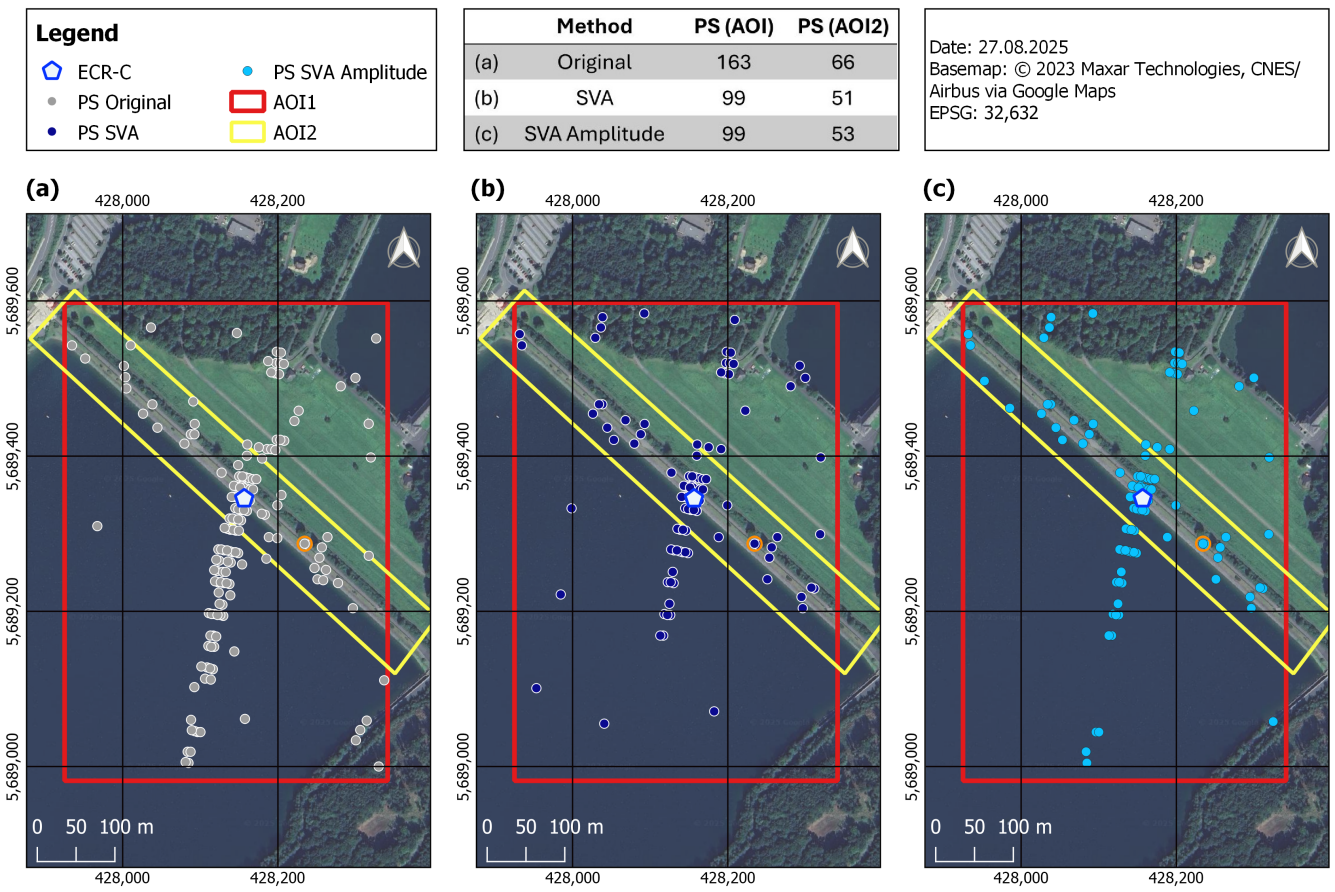
Comparison of PS points for Original (**a**), SVA_entireScene (**b**), and SVA_amplitudeBased (**c**) across the full time series. The PS point highlighted in orange marks the location of a validation PS. The table summarized the PS count for each method in both Area of Interests (AOIs).

**Figure 9:**
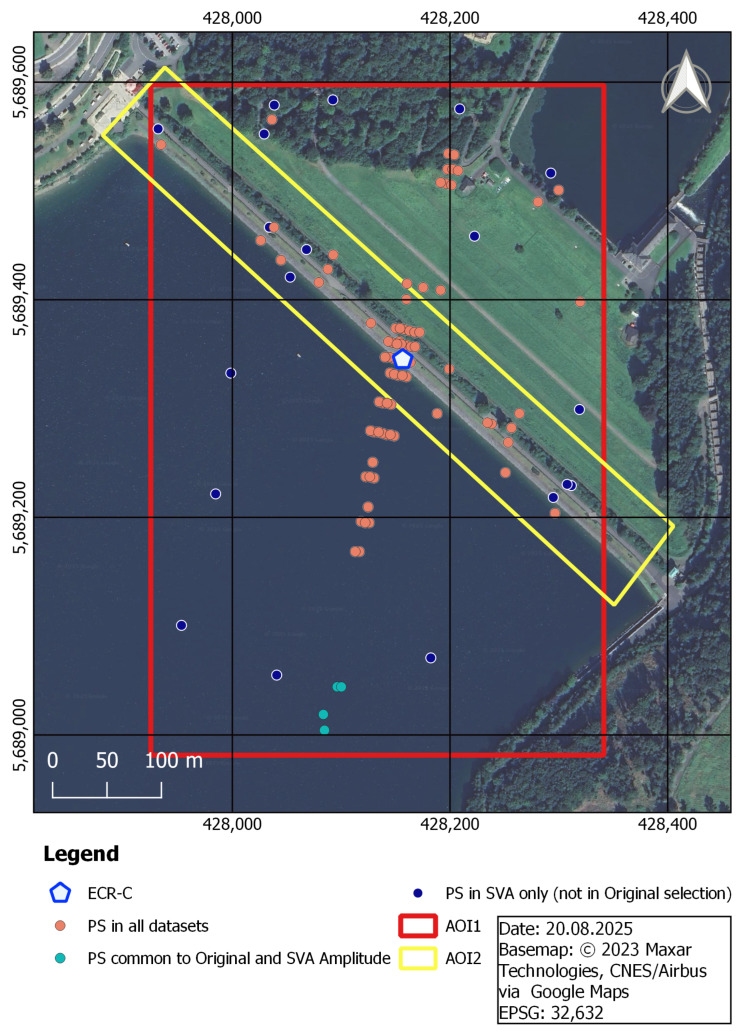
Overlay of PS points for Original, SVA_entireScene and SVA_amplitudeBased across the full time series.

The spatial distribution of PS points is shown in Figure 8. Both SVA_entireScene (b) and SVA_amplitudeBased (c) yielded fewer PS points than the Original (a) approach, with only minor differences in spatial distribution. The table embedded in the map summarizeed the total number of PS points detected within the two AOIs. Results for AOI1 (red) indicated that PS points over water were effectively removed by SVA_entireScene and SVA_amplitudeBased, while most PS points on the dam crest (AOI2, yellow) were preserved. Comparing SVA_entireScene and SVA_amplitudeBased, only minor differences were observed: SVA_amplitudeBased detected two additional PS points in AOI2 and exhibited slight visual variations in spatial distribution. As illustrated in Figure 9, most PS detected using SVA_entireScene and SVA_amplitudeBased were also included in the Original selection (orange). All PS points from SVA_amplitudeBased were also detected by the Original approach, whereas four PS points were not identified by SVA_entireScene (turquoise). Nineteen PS points were identified exclusively in the SVA_entireScene approach (dark blue). Nevertheless, the majority of PS points were common to both methods.

**Figure 10:**
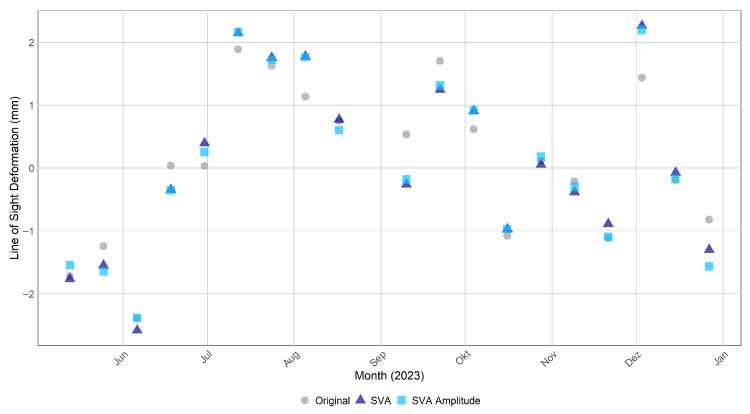
Temporal Deformation of the PS of the ECR-C signal after Persistent Scatterer Interferometry (PSI) Processing for Original (grey), SVA_entireScene (dark blue) and SVA_amplitudeBased (light blue) for the whole time series. The Root Mean Square Error (RMSE) between the Original and SVA_entireScene was 0.388 mm, and between the Original and SVA_amplitudeBased was 0.384 mm.

**Figure 11:**
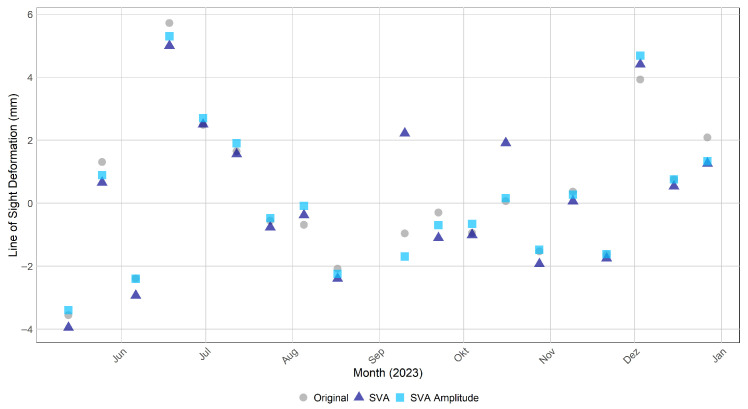
Temporal Deformation of a PS on the Sorpe crest [7.969463 51.35032] after PSI Processing for Original (grey), SVA_entireScene (dark blue) and SVA_amplitudeBased (light blue) for the whole time series. The RMSE between the Original and SVA_entireScene was 0.947 mm, and between the Original and SVA_amplitudeBased was 0.386 mm.

The PSI results were further analyzed by plotting the temporal deformation signal of the PS point representing the ECR-C signal, as shown in Figure 10. Both SVA variants, SVA_entireScene (dark blue) and SVA_amplitudeBased (light blue), differed slightly from the Original (grey), reaching deviations of up to 0.7 mm in September. The Root Mean Square Error (RMSE) between the Original and SVA_entireScene was 0.388 mm and between the Original and SVA_amplitudeBased 0.384 mm. The SVA_amplitudeBased temporal deformation pattern aligned more closely with the SVA_entireScene than the Original signal did. To further validate these results, an additional PS point at the crest of the Sorpe Dam was analyzed in Figure 11. The point’s location is illustrated in Figure 8, where the PS is circled in orange. The SVA_entireScene showed a larger deviation at this second location, reaching almost 3 mm in September, while the SVA_amplitudeBased method showed minimal deviation and remained close to the Original signal. The RMSE between the Original and SVA_entireScene was 0.947 mm and between Original and SVA_amplitudeBased 0.386 mm.

**Figure 12:**
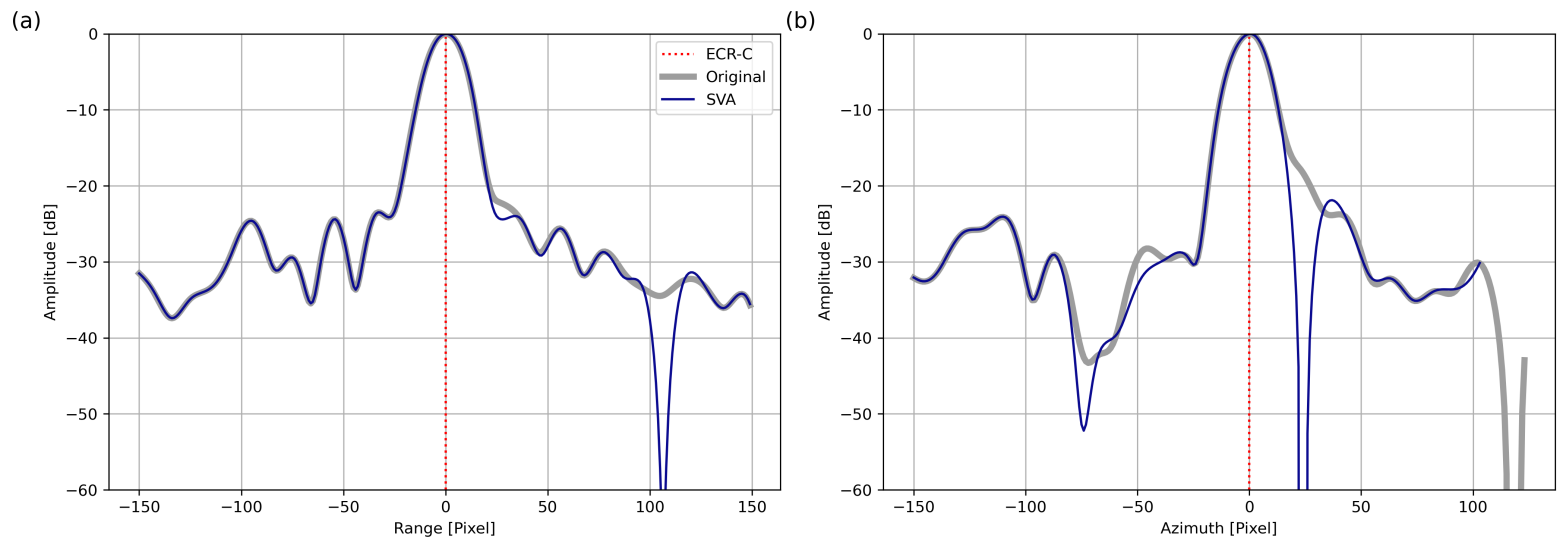
Comparison of the IPRs of the Amplitude for Original (grey) and SVA_entireScene (blue) versions in Range (**a**) and Azimuth (**b**), Secondary After Coregistration, 15 May 2023. The mean deviation between the Original and SVA_entireScene was 0.91 dB in Range and 4.37 dB in Azimuth.

To further validate the ECR-C signal, the IPR of the amplitude signal was analyzed for the Original and SVA_entireScene versions. The S-1 scene from 15 May 2023 was chosen, as it was the first in the time series. The amplitude was calculated from the complex SAR image using the I and Q components (see Section 3.4). Since the analysis was based solely on amplitude, the result of the SVA_amplitudeBased method was identical to that of the SVA_entireScene. For Original and SVA_entireScene, the brightest pixel, corresponding to the ECR-C signal, was identified, and a window of 150 pixels was extracted in both the azimuth and range directions. The 1D IPR profiles were oversampled by a factor of 10 using cubic interpolation. Edge values were handled implicitly by limiting interpolation to the valid index range without extrapolation. Amplitudes were stored and processed as 32-bit floating-point arrays. Profiles were normalized to their maximum value and converted to logarithmic scale (dB). The resulting IPR profiles, centered on the ECR-C peak (position 0), are shown in Figure 12 for the range and azimuth directions, highlighting differences in sidelobe reduction and mainlobe characteristics. In the range direction (a), the SVA_entireScene (dark blue) showed lower sidelobe levels around pixel 105, while it followed mostly the shape of the Original signal (grey). The mean deviation from the Original was 0.91 dB. In the azimuth direction (b), the differences became more pronounced. The SVA_entireScene deviated more at several positions, and its mainlobe appeared more distinct. Here, the mean deviation was 4.37 dB.

**Figure 13:**
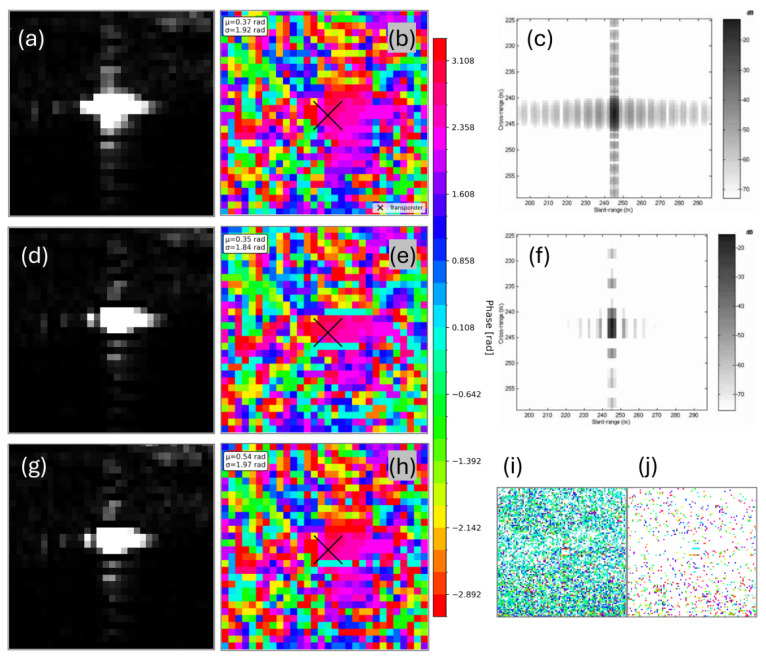
Comparison of amplitude and phase across Original, SVA_entireScene, and SVA_amplitudeBased and simulated SAR IPR. All amplitude and phase images corresponded to the interferogram computed for 15 May 2023 and were centered to the ECR-C’s location. (**a**) Amplitude image (Original), (**b**) Phase image (Original), (**c**) Simulated SAR IPR without SVA filter [24], (**d**) Amplitude image (SVA_entireScene), (**e**) Phase image (SVA_entireScene), (**f**) Simulated SAR IPR with SVA filter [24], (**g**) Amplitude image (SVA_amplitudeBased), (**h**) Phase image (SVA_amplitudeBased), (**i**) Phase difference: Original (**b**) minus SVA_entireScene (**e**), (**j**) Phase difference: Original (**b**) minus SVA_amplitudeBased (**h**).

A comprehensive overview of amplitude images, wrapped phase interferograms, pixel-wise phase differences, and a simulated IPR from Castillo-Rubio et al. (2007) [24] is shown in Figure 13. The interferogram from 15 May 2023 was chosen, as it was the first in the time series. Visually, both SVA methods SVA_entireScene (d) and SVA_amplitudeBased (g), exhibited a clear reduction in sidelobe intensity compared to the Original amplitude (a), consistent with the simulated sidelobe reduction illustrated in panels (c) and (f). The amplitude images from the SVA_entireScene (d) and the SVA_amplitudeBased (g) appeared identical, since both applied the same filtering to the amplitude component. With respect to phase, SVA_amplitudeBased (h) largely preserved the phase structure of the Original interferogram (b), showing only minor deviations. In contrast, SVA_entireScene (e) introduced more noticeable alterations in the phase pattern. To quantify these differences, the mean phase values (μ) and standard deviations (σ) were computed. The SVA_entireScene deviated by up to 0.02 rad from the Original mean, while the SVA_amplitudeBased showed a deviation of 0.17 rad. The standard deviation of phase differences between the Original (b) and SVA_entireScene (e) was 0.08 rad, whereas it was lower at 0.05 rad for the SVA_amplitudeBased (h). The phase difference map of the SVA_entireScene (i) revealed widespread pixel-level phase shifts, while the SVA_amplitudeBased difference map (j) showed only minor and sparsely distributed deviations. Deviation maps for the amplitudes were not included, as the amplitudes for SVA_entireScene (d) and SVA_amplitudeBased (g) were identical and would otherwise result in a blank (white) panel.

### 5.2. Interpretation of Results

The results demonstrate that the SVA_entireScene and SVA_amplitudeBased methods effectively reduce sidelobes compared to the Original approach by minimizing false PS detections, thereby enhancing PSI-based infrastructure monitoring at the Sorpe dam site. The novel SVA_amplitudeBased approach preserves the original phase, while the SVA filter operates on the amplitude to reduce sidelobe-affected pixels, ensuring more reliable dam monitoring in this case study. This is reflected in the PS point selection shown in Figure 8, where SVA_entireScene and SVA_amplitudeBased exhibit reduced sidelobe levels without loss of PS points on the dam crest (AOI2). This likely results from the method’s adaptive design, which adjusts the filtering strength based on local pixel gradients. In addition, its 2D application and separate processing of the I and Q components support effective sidelobe reduction across both spatial dimensions. SVA_amplitudeBased preserves PS selection more closely to the Original method than the SVA_entireScene approach (Figure 9). Since StaMPS PS selection relies on both amplitude and phase criteria, and the SVA_amplitudeBased approach retains the same phase as the Original, the resulting selection is nearly identical, with the difference that SVA_amplitudeBased is more selective than the Original approach.

Further, the IPR results (Figure 12) confirm that SVA achieves pronounced sidelobe reduction, especially in the azimuth direction at the Sorpe dam site, where the ECR-C generates a strong signal. The mainlobe is clearly separated from the sidelobes, and the sidelobe levels around the mainlobe are reduced both in dB and by a higher mean deviation compared to the range direction. The sharp drops observed in the SVA curve indicate that individual pixels are set to zero, corresponding to locations where the weighting factor $w_{u}$ falls within the range $0\leq w_{u}\leq \frac{1}{2}$, classifying them as sidelobes to be reduced. As a result, their amplitudes become zero and appear as steep nulls in the dB-scaled IPR. In this way, SVA reduces sidelobe energy while still preserving the mainlobe structure. However, after SVA filtering, the mainlobe of SVA_entireScene should be smaller and approximately 10 dB lower than that of Original. One possible explanation is that the ECR-C behaves as a non-ideal PS, either due to its strong artificial signal, highlighting the need for IPR data from other PS for comparison. Another explanation could be that the ECR-C is not perfectly aligned with the sidelobe direction. In addition, the S-1 data do not fulfill the optimal sampling condition required for SVA application as described by Fischer et al. (2006) (see Section 2.1). The method assumes an integer oversampling factor, which is not achieved in this case study and may contribute to the limited reduction of the mainlobe amplitude.

The simulated effect of SVA on SAR systems reported by Castillo-Rubio et al. (2007) [24] (Figure 13) aligns with the amplitude images obtained in this proof-of-concept case study. After applying SVA, the sidelobe energy is visibly reduced in both the simulation and the real-case amplitudes, while the amplitudes of SVA_entireScene and SVA_amplitudeBased are identical, their phases differ. The phase of SVA_amplitudeBased remains close to the Original, demonstrating that the amplitude-based approach operates by preserving the original phase while applying the SVA filter only to the amplitude. Despite its performance in sidelobe attenuation, the SVA_amplitudeBased application of SVA introduces minor deviations in phase-related outputs compared to the Original. These differences also appear in the deformation time series (Figure 10 and Figure 11). A likely explanation is that the method is not fully integrated into the SNAP processing environment, as the SVA filter is executed externally via Python. Although only the amplitude is modified and the original phase is preserved using Band Maths in SNAP, the external filtering step in Python may introduce subtle resampling, rounding, or interpolation effects during data export and reimport. To verify the magnitude of such effects, the Original dataset was processed twice under identical conditions and compared. The RMSE between the two runs of the PS of the ECR-C signal was found to be 0.13 mm, confirming that such minimal fluctuations occur at the level of machine precision and do not indicate any methodological error. At the same time, this value can be regarded as a reference noise level, demonstrating that sub-millimeter differences in the time series are within the expected numerical precision and do not reflect meaningful methodological variations. Regarding the RMSE, the PS lying on the dam crest shows that the SVA_amplitudeBased method aligns more closely with the Original signal than the SVA_entireScene (Figure 11). In contrast, the PS point corresponding to the ECR-C signal exhibits nearly identical RMSE values for both SVA approaches (Figure 10), suggesting that the ECR-C, due to its artificially strong backscatter, may not be the most suitable test case for evaluating subtle filtering effects. Overall, the results indicate that the SVA_amplitudeBased approach preserves the original phase information effectively, while both SVA_entireScene and SVA_amplitudeBased methods successfully reduce sidelobe-affected pixels compared to the Original approach in this scenario.

### 5.3. Comparison with Existing Studies

According to the literature, the SVA filter outputs in this study align with the findings of Stankwitz et al. (1994) [14], who observed that SVA achieves sidelobe reduction by operating directly on complex imagery [44]. In addition, Xiong et al. (2015) [45] reported similar findings, regarding cross-shaped sidelobes, noting that SVA reduces these patterns into more localized bright spots. However, many sidelobe structures remained visible after filtering, which is also the case in the present work. They proposed developed variants, as the standard SVA filtering effect was not strong enough to fully eliminate sidelobes as outlined in Section 2.1.

Furthermore, Fischer et al. (2006) [15] reported that SVA performed better in the azimuth direction than in the range direction, producing a sharper mainlobe response, which agrees with these results. As outlined in Section 2.1, Fischer et al. (2006) [15] described the optimal requirements for effective sidelobe reduction with SVA (rectangular spectral shape, Nyquist-rate or integer sampling, and absence of spectral shifts such as squint). The S-1 data used in this study are not specifically adjusted to meet these requirements. However, most of the necessary conditions are already satisfied. The observed sidelobes in the study area exhibit rectangular spectral support, forming a cross-shaped pattern along the x and y axes (see Figure 2). Additionally, the S-1 data are acquired in slant-range geometry, ensuring a constant sampling rate in the range direction, and are aligned in zero-Doppler geometry, which prevents spectral shifts due to squint, while these conditions support effective SVA application, S-1 data does not fulfill the optimal sampling rate condition described by Fischer et al. (2006) [15]. Several studies emphasized the importance of appropriate sampling for effective sidelobe reduction using SVA. Castillo-Rubio et al. (2007) [24] agreed that the sampling rate strongly influences the performance of sidelobe reduction algorithms. Deviations from the Nyquist criterion tend to degrade cancellation effectiveness due to insufficient frequency separation between the mainlobe and sidelobes. Zhai et al. (2007) [22] and Wang at al. (2012) [42] argued that deviations from integer sampling compromise the accuracy of sidelobe cancellation. In contrast, this study demonstrates that even without integer sampling, mainlobe separation and sidelobe reduction are achieved. This finding aligns with Wang at al. (2012) [42], who acknowledged that although SVA is less robust under non-integer sampling conditions, it can still effectively narrow the mainlobe and reduce sidelobes to some extent. These observations suggest that while SVA is capable of handling moderate deviations from ideal sampling, its reduction performance is maximized under proper sampling conditions.

Regarding the PSI aspects, the findings of Zhai et al. (2007) [22] showed that interferometric fringes become more clearly visible after applying SVA. In contrast, the present study does not show a improvement in fringe visibility, particularly in the vicinity of the ECR-C (Figure 13). The phase remains highly variable, and no pronounced stripe pattern is observed. Furthermore, Chaabane et al. (2009) [13] reported that all pixels selected by SVA are also identified as PS candidates in the original dataset without SVA filtering. However, SVA is more selective and effective at identifying stable PS points. In other words, all PS points detected by SVA are present in the original dataset, but not all original PS points are retained by SVA. This aligns with the findings of the SVA_amplitudeBased approach in the present study (Figure 9). In the study by Iglesias et al. (2013) [23], SVA improved PS selection. This is consistent with the findings of the present study, where most PS on the dam crest (AOI2: not affected by sidelobes) are retained, while PS detection is reduced over water, where sidelobes are present (Figure 8). However, Iglesias et al. (2013) [23] showed that using SVA-filtered images in the PS approach may overestimate amplitude dispersion, potentially leading to the loss of candidate pixels and negatively impacting phase quality. This concern is particularly relevant here, as the present study uses StaMPS for PSI processing, where PS selection is based on both amplitude dispersion and phase stability. As outlined in Section 4.2, StaMPS first identifies candidate PS using the amplitude dispersion index and then refines the selection through phase stability analysis, discarding decorrelated or noisy pixels [29]. To address the potential impact of SVA on amplitude-based selection, the present study investigates an amplitude-based approach that preserves the original phase information while modifying only the amplitude. Nevertheless, since amplitude is still affected, it continues to influence the PS selection.

**Figure 14:**
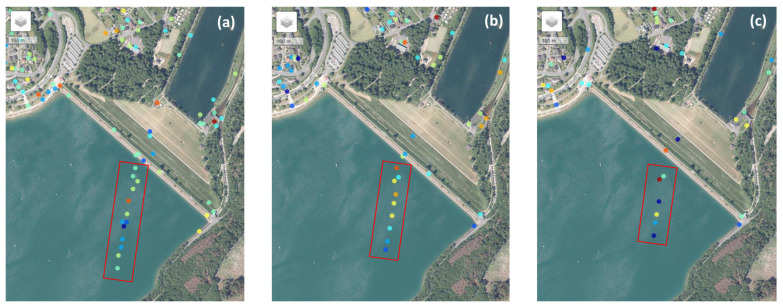
PS points affected by sidelobes for different time spans (PS colors are randomly assigned): (**a**) 12 months with 13 sidelobe-affected PS, (**b**) 18 months with 9 sidelobe-affected PS (−4), and (**c**) 24 months with 6 sidelobe-affected PS (−3). The red box highlights the location of the installed ECR-C (Sentinel-1A descending acquisitions, Orbit 139, Frame 421, start date: 1 January 2023, 12-day repeat cycle) [11]. Image: StaMPS-Visualizer via Leaflet [46], imagery source: ESRI [47]).

Overall, during PSI processing, incoherent signals tend to average out over time, while coherent targets remain detectable. Consequently, stable PS points, such as those on the dam crest, are preserved, whereas sidelobe-affected PS arising from incoherent scattering gradually vanish. Temporal decorrelation of incoherent SAR scattering therefore acts as a natural mechanism for sidelobe reduction as the observation period increases [3]. This effect is demonstrated in Figure 14, where sidelobe-affected PS diminish over time, and varying coherence levels lead to greater variability within the time series. Hence, longer observation periods may reduce or even eliminate the need for additional sidelobe reduction techniques during PSI processing.

### 5.4. Integration, Limitations and Perspectives

The integration of the SVA method into the SNAP2StaMPS workflow involves effort. Since SVA is not natively supported in SNAP, the algorithm must be implemented independently in Python and connected via the snappy interface. Setting up this interface is challenging due to strict compatibility requirements between Python and SNAP versions. However, once successfully configured, the workflow becomes relatively efficient: the SVA step is executed outside of SNAP in Python, and the output is returned in a compatible format, allowing processing to continue seamlessly within SNAP. Nonetheless, the amplitude-based SVA filtering approach remains particularly time-consuming. It requires storing both the SVA and non-SVA-filtered outputs to perform Band Maths in SNAP across the entire time series to recombine I and Q components and recover the original phase and SVA-filtered amplitude. The use of Band Maths is both labor-intensive and error-prone, as it is not automated and must be executed manually for each scene sequentially. This underlines the need for automation to improve efficiency and consistency, especially for large-scale datasets.

While the results of this case study demonstrate the potential of the developed method, their general applicability remains limited. The method is applied in a single, infrastructure-focused study area with a strong ECR-C signal and is only performed for descending passes. Whether the approach performs similarly in other environments, such as vegetated, or urban areas without ARs, and how it compares between ascending and descending passes requires further investigation. Another key consideration is the PS selection criteria used in StaMPS. Although the amplitude-based approach developed in this work preserves phase information, StaMPS selects PS points based on both amplitude and phase criteria. Alternative PSI software, such as the method proposed by Ferretti et al. (2001) [4], which relies solely on amplitude dispersion, may offer a more suitable framework for evaluating the performance of amplitude-based filtering strategies. Furthermore, StaMPS is primarily designed for natural terrain analysis and may not be fully optimized for infrastructure monitoring [29]. Nonetheless, its successful application for infrastructure monitoring has been demonstrated by Ziemer et al. [11], who compared PSI-processed S-1 time series with in situ pendulum measurements to validate the suitability for dam monitoring and reported good agreement.

Future research focuses on several key areas. First, sampling strategies for SVA should be refined to further enhance sidelobe reduction while maintaining phase fidelity. In addition, advanced SVA variants, such as MSVA (introduced in Section 2.1), should be tested to achieve enhanced sidelobe control. Incorporating alternative windowing techniques, such as applying a Hamming window within an otherwise identical processing pipeline, would demonstrate the benefits of the proposed method. For further comparison, using an SLRM would be informative, as it enables sidelobe effects to be evaluated without altering the complex SAR data. Second, the amplitude-based processing chain should be automated and integrated more tightly into platforms such as SNAP and workflows like SNAP2StaMPS. Additional case studies across varying land covers and structural types, including other dams in the Ruhrverband region, would help assess the robustness and generalizability of the method. Moreover, testing alternative PSI software that relies purely on amplitude criteria can provide clearer insight into the effectiveness of the amplitude-based approach. Amplitude dispersion and temporal coherence at each StaMPS step could also provide additional insight. Future work also aims to quantify the impact of sidelobe reduction on phase quality, coherence, and deformation accuracy, contributing to the development of algorithms that balance sidelobe reduction with reliable phase preservation. In this context, evaluating metrics such as the Peak Sidelobe Ratio (PSLR), the Integrated Sidelobe Ratio (ISLR), and the −3 dB main-lobe widths in both range and azimuth would provide a robust assessment of sidelobe behaviour for SVA in comparison with other sidelobe reduction techniques. Beyond algorithmic improvements, hardware and system-level advancements play a critical role in minimizing sidelobe artifacts. Enhancements in antenna design, beam steering, and signal processing reduce sidelobe generation at the source, thereby improving data quality prior to post-processing. In addition, the placement and configuration of ARs should be optimized to avoid interference with natural scatterers. Advanced AR systems allow internal delay adjustments, enabling deliberate placement of the AR signature in less critical regions of the SAR image. Future systems prioritize such flexibility while maintaining ease of configuration. Finally, integrating sidelobe reduction into standardized PSI workflows requires a multi-faceted approach, combining algorithmic filtering, hardware design, AR management, and software-level automation. A comprehensive and adaptable strategy is needed to effectively address the challenge of sidelobes in high-precision SAR applications such as infrastructure monitoring, long-term deformation measuring, and structural health analysis.

## 6. Conclusions

The study examined sidelobe reduction using the SVA filter at the Sorpe Dam. Strong backscatter from an ECR-C caused false PS candidates during the PSI processing of the descending S-1 time series. The objective was to enhance dam monitoring by reducing sidelobe-affected pixels during preprocessing. The SVA filter was implemented within the SNAP2StaMPS workflow using SNAP, Python, and the snappy interface for subsequent PSI processing in StaMPS. In addition, the amplitude-based SVA approach developed in this work recombined the original phase from the Cartesian representations of I and Q after coregistration using the original and SVA-filtered SAR images, thereby achieving amplitude-based sidelobe reduction while preserving the original phase.

The results showed that both SVA_entireScene and SVA_amplitudeBased effectively reduced sidelobes at the dam site, with the number of sidelobe-affected PS decreasing by 39.26% after SVA filtering. Deformation estimates remained consistent with the Original, yielding an RMSE of approximately 0.38 mm for both approaches. The amplitude images visually demonstrated the reduction of sidelobe energy in the SAR images, while SVA achieved successful mainlobe–sidelobe separation, sidelobe reduction was more effective in the azimuth than in the range direction, as confirmed by the IPR, achieving sidelobe levels of around −22 dB. The results further indicated that the SVA_amplitudeBased difference map showed only minor and sparsely distributed deviations, confirming that the original phase was preserved. This was also reflected in the PS distribution, while SVA_amplitudeBased retained all PS points detected by the Original approach and removed sidelobe-affected PS, the PS distribution of SVA_entireScene differed from the Original by nineteen PS points, introducing new PS points.

Overall, the findings demonstrate that the SVA_amplitudeBased approach outperforms the SVA_entireScene method, as it effectively reduces sidelobe-affected pixels while preserving the original phase, making it especially valuable for PSI-based monitoring in areas with strong sidelobes, such as the Sorpe Dam site. This study also provides a basis for integrating the SVA filter for sidelobe reduction into PSI workflows such as SNAP2StaMPS and emphasizes the potential benefit of preserving phase information when reducing sidelobe effects in this specific scenario. Future research should focus on automating the amplitude-based processing chain, testing the method in diverse environments, and quantifying the impact of sidelobe reduction on phase quality and deformation accuracy, thereby ensuring broader applicability for infrastructure monitoring and long-term deformation analysis.

## Data Availability

The Sentinel-1 SAR data used in this study are publicly available from the Alaska Satellite Facility (2025) [33]. The 1-arc-second global DEM from the SRTM mission is available through the ESA SNAP toolbox. The SNAP2StaMPS workflow is available on GitHub (Blasco & Fourmelis (2021)) [36]. The StaMPS software is available on GitHub (Hooper et al. (2018)) [29], and the custom Python implementation of the SVA filtering developed for this study is available on GitHub (Liedel (2025)) [43].

## Abbreviations

The following abbreviations are used in this manuscript:

2D	two-dimensional
AOI	Area of Interest
AR	Active Reflector
CDA	Complex Dual Apodization
CR	Corner Reflector
DA	Dual-Apodization
db	decibel
DEM	Digital Elevation Model
ECR-C	Electronic Corner Reflector—C band
ESA	European Space Agency
GHz	Gigahertz
GSVA	General Spatially Variant Apodization
I	In-phase
InSAR	Synthetic Aperture Radar Interferometry
IPR	Impulse Response
ISLR	Integrated Sidelobe Ratio
IW	Interferometric Wide Swath
LOS	Line of Sight
MATLAB	MATrix LABoratory
MHz	Megahertz
MSVA	Modified Spatially Variant Apodization
NASA	National Aeronautics and Space Administration
PS	Persistent Scatterer
PSI	Persistent Scatterer Interferometry
PSLR	Peak Sidelobe Ratio
Q	Quadrature
RMSE	Root Mean Square Error
RSVA	Robust Spatially Variant Apodization
S-1	Sentinel-1
SAR	Synthetic Aperture Radar
SLC	Single Look Complex
SLRM	Side-Lobe Risk Map
SNAP	SentiNel Application Platform
SNAP2StaMPS	SentiNel Application Platform to Stanford Method for Persistent Scatterers
SNAPHU	Statistical-Cost Network Flow Algorithm for Phase Unwrapping
snappy	SNAP-Python
SRTM	Shuttle Radar Topography Mission
StaMPS	Stanford Method for Persistent Scatterers
STEP	Scientific Toolbox Exploitation Platform
SVA	Spatially Variant Apodization
TOPSAR	Terrain Observation by Progressive Scans Synthetic Aperture Radar
TRAIN	Toolbox for Reducing Atmospheric InSAR Noise

## Appendix A

**Table A1:** SNAP2StaMPS Workflow—Parameter Settings.

Parameter	Value
Interferometric Wide Swath (IW)	IW2
Number of Bursts	5–7
Polarization	Vertical-Vertical (VV)
Reference Scene	29 August 2023
Area of Interest (AOI)	Lon: 7.826307°–8.133924°, Lat: 51.263001°–51.431122°

**Table A2:** StaMPS Workflow—Parameter Settings for Sentinel-1 Data and Sorpe Dam Case Study [11,29].

Parameter	Default	Selected	Description
**Preprocessing Settings**			
mt_prep_snap		0.4	Amplitude dispersion threshold to exclude pixels with high amplitude variability
**Processing Settings**			
density_rand	20	100	Increases density of randomly selected pixels for initial phase stability estimation
max_topo_err	20	5	Limits pixels with high DEM error, improving accuracy in geometric phase correction
scla_deramp	‘n’	‘y’	Subtracts phase ramp for local signal analysis
weed_standard_dev	1	1.2	Threshold for phase noise standard deviation to refine PS selection by removing noisy pixels
select_reest_gamma_flag	‘y’	‘n’	Saves processing time by skipping re-estimation
**Case Study Settings**			
ref_centre_lonlat	[0 0]	[7.968285 51.350979]	Set circular reference area (circle centre)
ref_radius	Inf	500	Set circular reference area (circle radius)
